# *cis*-Regulatory Complexity within a Large Non-Coding Region in the *Drosophila* Genome

**DOI:** 10.1371/journal.pone.0060137

**Published:** 2013-04-22

**Authors:** Mukta Kundu, Alexander Kuzin, Tzu-Yang Lin, Chi-Hon Lee, Thomas Brody, Ward F. Odenwald

**Affiliations:** 1 Neural Cell-Fate Determinants Section, National Institute of Neurological Disorders and Stroke, National Institutes of Health, Bethesda, Maryland, United States of America; 2 Section on Neuronal Connectivity, National Institute of Child Health and Human Development, National Institutes of Health, Bethesda, Maryland, United States of America; University of Iceland, Iceland

## Abstract

Analysis of *cis*-regulatory enhancers has revealed that they consist of clustered blocks of highly conserved sequences. Although most characterized enhancers reside near their target genes, a growing number of studies have shown that enhancers located over 50 kb from their minimal promoter(s) are required for appropriate gene expression and many of these ‘long-range’ enhancers are found in genomic regions that are devoid of identified exons. To gain insight into the complexity of *Drosophila cis-*regulatory sequences within exon-poor regions, we have undertaken an evolutionary analysis of 39 of these regions located throughout the genome. This survey revealed that within these genomic expanses, clusters of conserved sequence blocks (CSBs) are positioned once every 1.1 kb, on average, and that a typical cluster contains multiple (5 to 30 or more) CSBs that have been maintained for at least 190 *My* of evolutionary divergence. As an initial step toward assessing the *cis*-regulatory activity of conserved clusters within gene-free genomic expanses, we have tested the *in-vivo* enhancer activity of 19 consecutive CSB clusters located in the middle of a 115 kb gene-poor region on the 3^rd^ chromosome. Our studies revealed that each cluster functions independently as a specific spatial/temporal enhancer. In total, the enhancers possess a diversity of regulatory functions, including dynamically activating expression in defined patterns within subsets of cells in discrete regions of the embryo, larvae and/or adult. We also observed that many of the enhancers are multifunctional–that is, they activate expression during multiple developmental stages. By extending these results to the rest of the *Drosophila* genome, which contains over 70,000 non-coding CSB clusters, we suggest that most function as enhancers.

## Introduction

Transcriptional activity of dynamically expressed genes is controlled in part by multiple pattern-specific enhancers that regulate different aspects of a gene's complete spatial/temporal expression, and many of these enhancers are clustered close to their regulated genes (for examples see [1]–[4]). A survey of *cis*-regulatory DNA surrounding developmental genes indicates that the *Drosophila* genome may harbor more than 50,000 enhancers [5]. Analysis of chromosome DNAse-1 hypersensitivity profiles suggests that many genes that are active during embryonic development use multiple enhancers [6]. Other surveys have revealed that transcription factor (TF) high occupancy target regions map to active embryonic enhancers located close to structural genes [7]. These studies have also shown that most enhancers are functionally autonomous, since they correctly regulate heterologous transgene expression dynamics outside of their endogenous chromosomal environment (reviewed by [8] [9]).

Phylogenetic footprinting of vertebrate, *Drosophila* or nematode genomic DNA has revealed that enhancers can be distinguished from other essential gene regions based on their characteristic pattern of conserved sequences [2] [4] [10]–[14]. Collectively, these studies have shown that most enhancers are made up of clusters of 5 to 30 or more conserved sequence blocks (CSBs) [15]–[17]. On average, enhancer CSB clusters span ∼1 kb and are flanked by non-conserved DNA of variable length. Self-alignment of conserved sequences within enhancers reveals that their CSBs contain repeat and palindrome sequence elements that make up, on average, over 60% of their sequences [17]. TF DNA-binding site searches of characterized enhancers reveals that while most CSBs contain core docking sites for known TFs, much of their conserved sequences consists of novel repeat, palindromic or single copy sequence elements. Genome-wide systematic manual curation of conserved *Drosophila* DNA has identified over 70,000 non-coding conserved sequence clusters [17]. Thus far, studies that have tested individual clusters closely associated with *Drosophila* developmental genes, such as *nerfin-1* [2], *hunchback* [18], *sloppy-paired* [3] and *castor* [4], have all shown that each cluster is an enhancer and many enhancers are multifunctional in that they regulate embryonic and/or adult developmental gene expression.

While examination of gene neighborhoods reveals that most non-coding regions associated with developmental determinants contain *cis*-regulatory sequences [5], sequence conservation tracks that span the fly genome [19] reveal that clusters of conserved sequences are not exclusively restricted to gene neighborhoods. Large gene-free regions termed 'gene deserts' in vertebrates are thought to consist of reservoirs of enhancers that function at a distance to regulate gene expression [20] [21]. For example, a 645 kb region that separates the vertebrate Iroquois genes *Irx3* and *Irx5* contains multiple Iroquois-specific enhancers [22]. Likewise, in *Drosophila* a distal non-coding region adjacent to the *Drosophila iroquois* gene complex contains multiple enhancers required for wild-type *irx* gene expression [23]. In addition, analysis of *cut* gene regulation has identified one of its wing margin enhancers 80 kb away from its proximal promoter region [24]. Other studies have located *invected* gene *cis*-regulatory elements positioned ∼78 kb upstream of its transcribed sequence [25] and remote shadow enhancers have been described for *shavenbaby*, a transcript of the *ovo* locus [26].

To what extent do clustered CSBs that reside in gene-free genomic expanses function as *cis*-regulatory enhancers? As an initial step toward addressing this question, we have examined the frequency of conserved sequence clusters and their CSB composition within 39 different *Drosophila* regions lacking exons that are greater than 65 kb in length, located throughout the genome. Comparative analysis of orthologous regions in other *Drosophila* species reveals that within these non-coding regions, such clusters occur on average once every 1.1 kb and, similar to known enhancers, each consists of multiple CSBs that contain unique sets of repeat and palindromic elements. Our comparative analysis also reveals that, like characterized enhancers, subsets of adjacent CSBs clustered within exon-poor regions also form ‘super-blocks:’ regions of invariantly spaced CSBs.

To address the function of ‘gene-distant’ CSB clusters, we tested the *cis*-regulatory function(s) of consecutive clusters within one gene-free 115 kb expanse that contains 90 conserved clusters located on the 3^rd^ chromosome between the *vvl* and *Prat2* genes. Earlier studies have identified tracheal and neural enhancers within this region [17] [27]. Our *in vivo* enhancer-reporter studies on 19 consecutive clusters demonstrate that each functions as an enhancer that regulates reporter expression in defined sets of tissues and developmental phases, thus representing a remarkable diversity of expression patterns. Many of these enhancers have proven to be multifunctional, directing expression dynamics at different times during development and in different cell types. Based on these findings, we estimate that the fly genome may contain over 70,000 enhancers and many are likely to be multifunctional.

## Results

### Sequence conservation within *Drosophila* gene-poor genomic expanses

**Figure 1:**
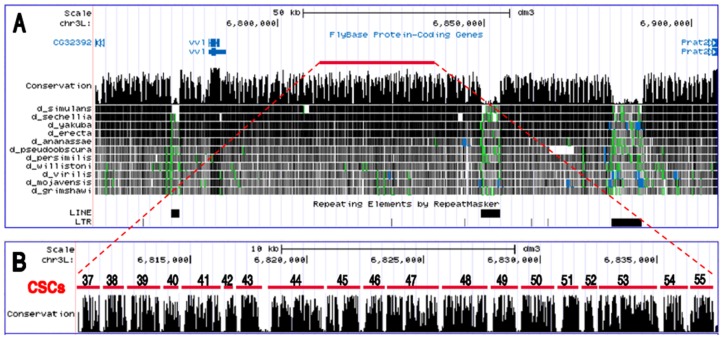
DNA conservation spanning the *Drosophila vvl* locus. (A) Shown is an UCSC Genome Browser view covering 150 kb of DNA located on the left arm of the *D. melanogaster* 3^rd^ chromosome (http://genome.ucsc.edu). The linear representation includes the *vvl* transcribed region and flanking DNA starting from the upstream neighboring *CG32392* gene and extending to the 3′ end of the downstream *Prat2* transcribed sequence. A 12 species *Drosophila* DNA conservation track [19] reveals the presence of conserved sequence clusters throughout the locus. The red bar (positioned 30 kb downstream of the *vvl* transcribed sequence) covers the 27 kb of the non-coding intergenic region that was examined in this study for the presence of independent *cis*-regulatory enhancers. Aligned below the conservation track are identified Line and LTR repeat elements present within the *D. melanogaster* DNA that are not present in the same orthologous positions within many of the other species included in the conservation analysis. (B) An expanded view of the intergenic region studied for its *cis*-regulatory activity (highlighted in panel A) reveals 19 consecutive conserved sequence clusters that were independently tested for their *cis*-regulatory activity. Cluster numbers correspond to their designation in the *cis*-Decoder *D. melanogaster* genome-wide sequence conservation database [17].

**Figure 2:**
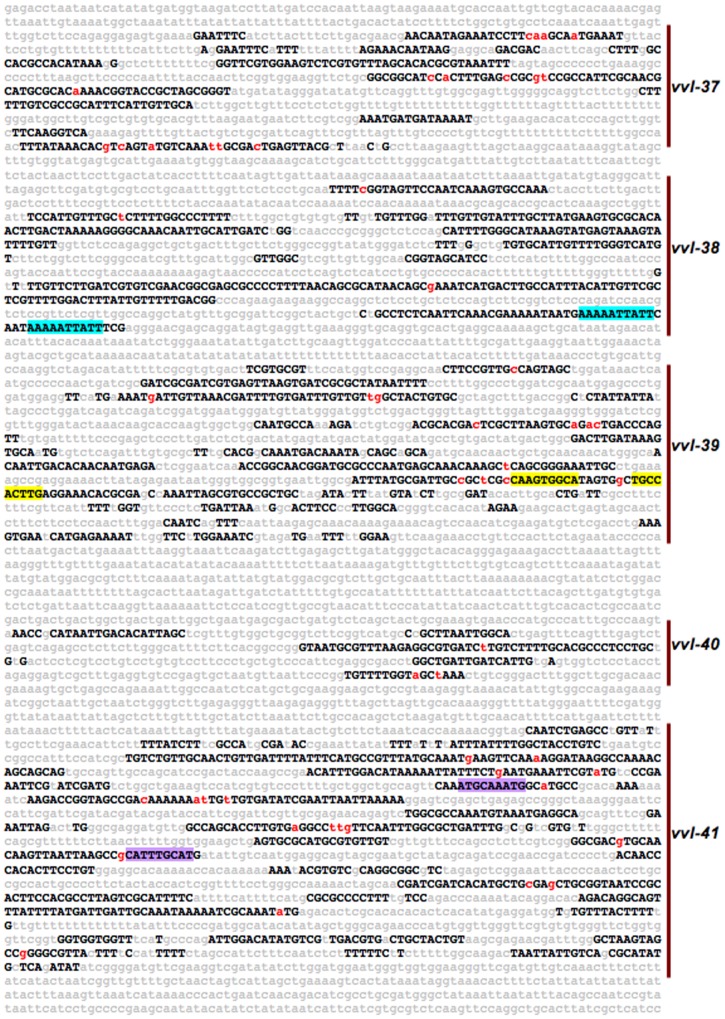
Gene-distant conserved sequence clusters are made up of multiple conserved sequence blocks. Shown, is a *D. melanogaster* relaxed *EvoPrint* spanning the first (most 5′) 6,351 bp of the *vvl* 3′ flanking intergenic region that includes the conserved sequence block (CSB) clusters *vvl-37* through *vvl-41* (indicated by vertical bars in left margin). CSB clusters are resolved by their flanking less-conserved inter-cluster sequences of 150 or more bp. Capital letters represent bases in the *D. melanogaster* reference sequence that are conserved in all, or all but one, of the orthologous regions within the *D. simulans*, *D. sechellia*, *D. erecta*, *D. yakuba*, *D. ananassae*, *D. pseudoobscura*, *D. persimilis*, *D. willistoni*, *D. virilis*, *D. mojavensis* and *D. grimshawi* genomes. Less or non-conserved DNA is shown as lower case gray letters and the lower-case red-font bases indicate invariant spacer length DNA between CSBs. Colored highlighted conserved sequences within the *vvl-38* (blue), *vvl-39* (yellow), and *vvl-41* (purple) clusters represent repeat elements that are discussed in Text S1.

As an initial step toward assessing the frequency and diversity of long-range enhancers in the *Drosophila* genome, we documented the occurrence of CSB clusters within 39 genomic expanses that each span at least 65 kb and are devoid of known or predicted protein encoding sequences (Table 1). The largest of these regions was an expanse of non-coding sequence of ∼142 kb associated with the *Antp* gene. None of the gene-poor regions are as large as those in mammals; it is known that the *Drosophila* genome is approximately an order of magnitude more compact, and the density of conserved elements is greater in *Drosophila* [19]. Also included in the phylogenetic footprinting survey were sequences in intragenic regions within large introns. *EvoPrints* covering these non-coding regions located throughout the genome (covering a total of ∼3.26 Mb or ∼2.7% of the euchromatic genome) revealed a near uniform conserved sequence cluster density of one per every ∼1.1 kb even in regions located over 50 kb from transcribed sequences (see Materials and Methods for *EvoPrint* conditions and [17] for database details). Clusters of CSBs were defined as independent when they were separated by at least 150 bp of non-conserved sequences and were resolved with *EvoPrint* conditions that represented a cumulative evolutionary divergence (the length of time that multiple species have evolved separately from one another) in excess of 190 million years. When compared to similar clusters close to transcriptional start sites, we did not detect significant differences between their genome density/spacing, their average number of CSBs, or differences in the degree of evolutionary sequence conservation of their CSBs (Figures 1, 2, S1, S2, S3, S4, and data not shown).

**Table 1:** *D. melanogaster* CSB clusters within non-coding regions >65 kb.

Chromosome Location	Flanking Genes (5′ – 3′)	Length (kb)	^#^ of CSCs
chr2R:2,416,250–2,487,059	jing^→^–CG15233^←^ *	70.8	63
chr2R:4,156,848–4,234,973	CG30371^←^–Pdm3^→^	78.1	70
chr2R:10,944,185–11,025,474	CG33467^←^–chn^→^	81.3	59
chr2R:15,893,693–15,999,491	CG16898^←^–18 w^→^	105.8	89
chr2R:16,243,641–16,316,603	CG11192^←^–CG12484^→^	73.0	57
chr2R:17,300,726–17,367,629	Sdc^←^–Sdc^←^	67.0	73
chr2L:1,614,340–1,690,699	RFeSP–chinmo	76.4	65
chr2L:8,564,841–8,631,270	Sema-1a^→^–Sema-1a^→^	66.4	53
chr2L:12,822,253–12,911,000	Kek1^←^–ACXC^→^	88.7	88
chr2L:17,827,895–17,914,722	CadN2^←^–CG43271^←^	86.8	85
chr2L:20,970,616–21,052,985	CG42238^←^–betaInt-nu^→^	82.4	66
chr3L:5,013,564–5,097,509	CG12027^←^–CG34047^←^	83.9	68
chr3L:5,269,532–5,337,221	Shep^←^–Lama^←^	65.6	61
chr3L:6,788,236–6,903,573	vvl^→^–Prat2^←^	115.3	90
chr3L:10,691,176–10,765,559	NijA^←^–CG43245^→^	74.4	78
chr3L:13,658,522–13,771,330	bru3^←^–CG34243^←^	112.8	98
chr3L:15,335,752–15,401,048	Toll6^→^–CG33259^→^	65.3	76
chr3L:15,722,537–15,801,891	Comm^←^–CG6244^→^	79.4	66
chr3L:18,297,975–18,389,946	grim^←^–rpr^←^	92.0	99
chr3L:19,140,122–19,229,706	Fz2^←^–CG33647^→^	89.6	86
chr3L:21,835,505–21,900,787	CG14563^←^–mub^→^	65.3	50
chr3L:22,076,308–22,158,687	msopa^→^–Olf413^→^	99.2	86
chr3R:814,412–910,094	CG2022^←^–corto^→^	95.7	58
chr3R:2,735,634–2,878,317	Antp^←^–Sod-1^→^	142.6	140
chr3R:4,269,254–4,335,814	PQBP-1^←^–OR85b^←^	66.6	57
chr3R:6,262,387–6,337,098	Cyp12e1^←^–hth^←^	74.7	63
chr3R:7,096,987–7,172,245	CG31386^←^–KP78b^←^	75.3	63
chr3R:10,258,903–10,335,707	cv-c^←^–HtrA1^←^	76.8	70
chr3R:10,747,590–10,840,491	CG3837^←^–CG14861^→^	92.9	92
chr3R:11,378,494–11458604	CG18516^←^–CG5302^→^	80.1	65
chr3R:18,671,035–18,741,129	CG4704^←^–klg^→^	70.1	66
chr3R:19,238,330–19,307,587	CG4374^←^–CG31225^→^	69.3	52
chr3R:21,226,341–21,299,237	CG31439^←^–CG5127^→^	72.9	70
chr3R:24,179,225–24,287,776	Or98b^→^–beat-VI^→^	108.6	97
chrX:972,189–1,039,710	CG3655^←^–CG14626^→^	67.5	62
chrX:3,866,525–3,971,906	CG6414^←^–CG32790^→^	105.4	83
chrX:7,004,130–7,091,461	fz4^←^–CG9650^→^	87.3	75
chrX:16,018,498–16,105,580	disco-r^←^–disco^←^	87.1	72
chrX:17,218,418–17,291,800	B-H2^→^–BH-1^→^	73.4	73

* Arrows indicate direction of transcription.

**Figure 3:**
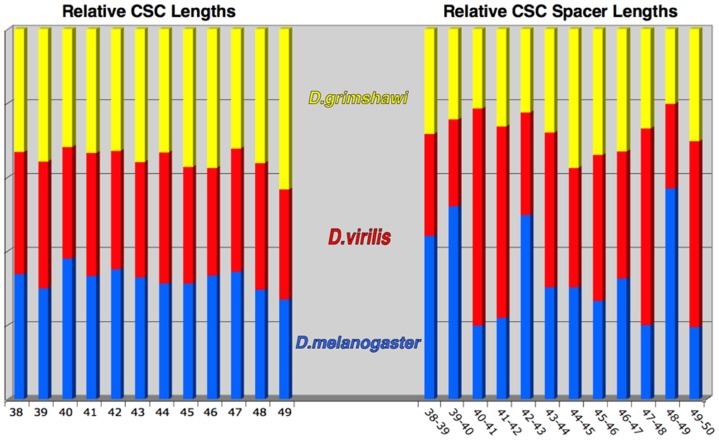
Evolutionary constraints on CSB cluster structure. Multi-species analysis of CSB clusters and their flanking spacer regions reveals that the less-conserved spacer DNA has greater evolutionary sequence length variability when compared to their flanking CSB clusters. Shown, are percentage base pair length differences between *D. melanogaster* (blue) *D. virilis* (red) and *D. grimshawi* (yellow) *vvl* clusters 38 through 49 and the percent differences within their flanking spacer regions (each column represents 100%).

A prominent feature of both gene-proximal and -distal conserved clusters is that the intervening non-conserved spacer regions between adjacent clusters have greater sequence length variability among different species in comparison to regions within clusters [2]. A graphic representation of this is shown in Figure 3. The lower sequence length variability within clusters indicates that there are differences in the structural constraints within clusters in contrast to inter-clustal flanking sequences. Pair-wise alignments of clustered CSBs among different drosophilids reveal that in many cases spacing between adjacent CSBs is not variable (Figure 2; and data not shown). We refer to neighboring CSBs that are separated by a conserved spacer length as a ‘super-block’ and suggest that inherent structural requirements for enhancer function place evolutionary constraints on occurrence of indels between CSBs within a super-block. There are, however, species-specific exceptions to the sequence length constraints observed between CSBs, and the variability observed in an individual species or in a subset of species is informative with regard to functional compartmentalization within an enhancer (see below).

### *cis*-Regulatory analysis of consecutive CSB clusters within a gene-poor region

Many of the non-coding regions examined in this study are flanked by developmentally regulated genes. For example, a 115 kb non-coding expanse separates the *ventral veins lacking* (*vvl*) gene, encoding a POU-domain containing transcription factor, and the Phosphoribosylamidotransferase-2 (*Prat2*) gene (Figure 1a). *EvoPrint* analysis of this region identifies 90 conserved sequence clusters and each contains CSBs that have been conserved for more than 190 million years of cumulative evolutionary divergence (Figures 2 and S1, S2, S3, S4; and data not shown). Both *vvl* and *Prat2* functions are required during multiple phases of development and in a variety of cell types. For example, *vvl* function is required for the correct migration of tracheal cells and glia during embryonic development [28], specification of motor neuron identity in the embryonic ventral cord [29], correct development of the peripheral nervous system [30], embryonic brain neural precursor cell identity [31], development of adult external mechanosensory organs [32], dendritic targeting of olfactory projection neurons in adults [33], [34], the correct temporal identity of optic lobe neurons [35] and for the development of wing imaginal disc cells [36]. In addition, *vvl* may also be required for correct epidermal development, as it is dynamically expressed in the epidermis during embryonic development [37]. Prat2 is expressed in embryos and larvae, as well as in testis [38], and in situ mRNA localization studies reveal expression in the embryonic yolk nuclei [39]. Prat2 is also required for metamorphosis during pupal development [39].

**Figure 4:**
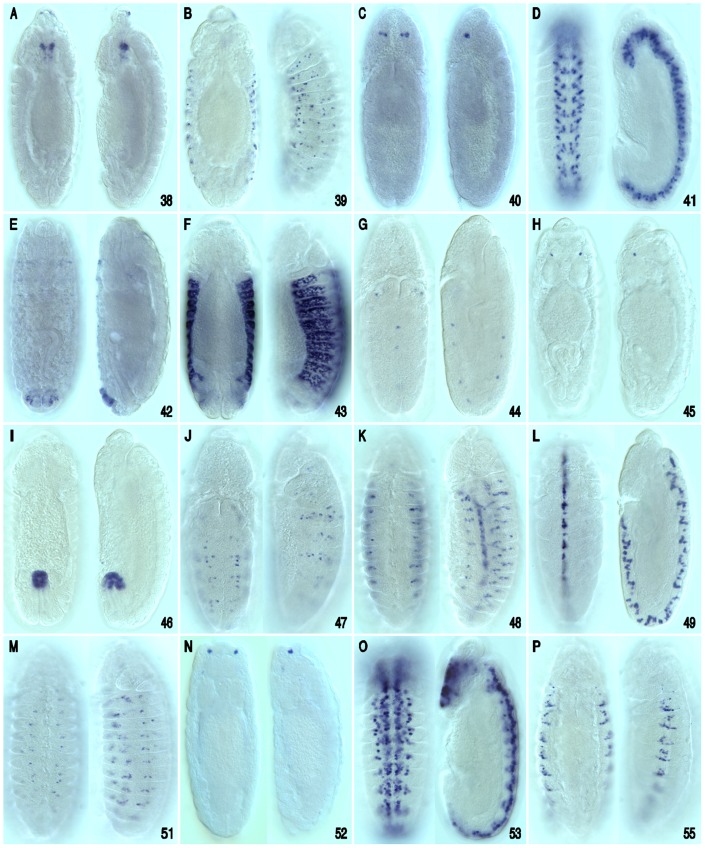
Conserved cluster *cis*-regulatory enhancer activity during embryonic development. Enhancer/reporter transgene expression analysis during embryonic development reveals that many of the tested CSB clusters are functionally independent embryonic enhancers that direct expression in different spatial/temporal patterns within the developing embryo. Shown are enhancer-reporter embryo expression patterns for 16 of the 19 consecutive clusters tested. Whole-mount mRNA stained embryos (staging according to Hartenstein and Campos-Ortega [58]; dorsal or ventral views adjacent to lateral views are shown for each cluster-reporter transgene; anterior up) to reveal peak reporter mRNA expression detected by a digoxigenin labeled Gal4 riboprobe for each of the cluster/enhancer-reporter constructs. The numbers in the lower right corner of each panel correspond to the clusters shown in Figure 1 and Figures S1, S2, S3, S4 and described in Table 2. (A) Dorsal and lateral view of a stage 13 embryo. *vvl-38* activates transgene reporter expression in a small cluster of cells within or near the developing antenno-maxillary complex and within a cluster of anterior gut epidermal cells positioned adjacent to the cephalic lobes. (B) Dorsal and lateral surface views of a stage 13 embryo. *vvl-39* drives expression in putative PNS cells. (C) Dorsal and lateral views of a stage 10 embryo. *vvl-40* activates expression in two adjacent NBs within each cephalic brain lobe. (D) ventral and lateral view of a stage 11 embryo. *vvl-41* drives expression in a set of NBs after they have generated their first GMC progeny. (E) Dorsal and lateral view of a stage 15 embryo. *vvl-42* drives expression in cells of the gut ectoderm. (F) Dorsal and later surface view of stage 13 embryo. *vvl-43* drives expression in late lateral ectodermal cells. (G) Dorsal and lateral view of a stage 10 embryo. *vvl-44* drives expression in a single midline cell per segment and in segmentally repeated lateral cells, possibly PNS cells. (H) Dorsal and lateral view of a stage 14 embryo. *vvl-45* drives expression in a bilateral pair of brain neurons. (I) Dorsal and dorsal-lateral views of a stage 13 embryo. *vvl-46* drives expression in the posterior midgut. (J) Dorsal and lateral view of a stage 11 embryo *vvl-47* drives in a few unidentified cells per hemisegment in the neuroectoderm and CNS. (K) Deep ventral and lateral view of a stage 10 embryo. *vvl-48* drives expression in segmentally repeated clusters that appear to be tracheal placodes. (L) Ventral and lateral view of stage 11 embryo. The *vvl-49* cluster activates reporter expression in ventral cord midline glial cells (also shown in Figure 8). (M) Ventral and lateral view of a stage 13 embryo. *vvl-51* drives expression in segmentally repeated putative neurons in the peripheral nervous system. (N) Dorsal and lateral views of a stage 14 embryo. The *vvl-52* cluster activates reporter expression in two bilaterally symmetrical cells within the antenno-maxillary complex. (O) Ventral and lateral views of a stage 12 embryo. *vvl-53* cluster drives expression in CNS NBs (both brain and ventral cord) during late NB linage development. (P) Ventral and lateral surface views of a stage 14 embryo. *vvl-55* activates expression in cells that line tracheal branches.

**Figure 5:**
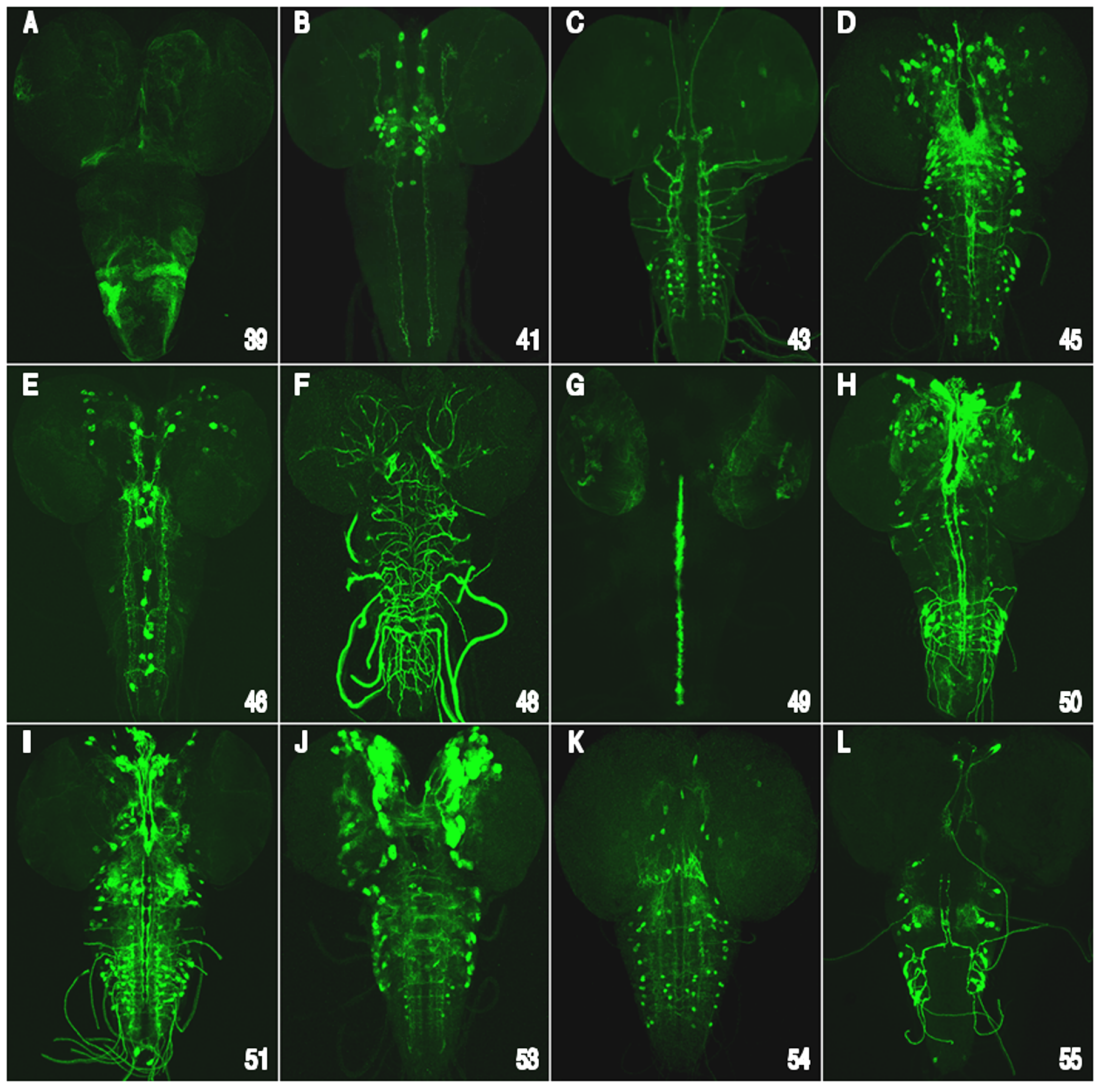
Expression of enhancer/reporter transgenes in the larval CNS. (A–L) During 3^rd^-instar larval development, most enhancer-*Gal4* transgenes from *vvl-37* to *vvl-55* (twelve are illustrated) activate UAS/GFP-CD8 tagged reporter expression in neural precursors, neurons or glia within sub-regions of the cephalic lobes and in the thoracic ventral cord. Shown are stacked images of dorsal views of dissected CNS preparations from wandering third-instar larva (anterior up). (A) *vvl-39* activates reporter expression in a subset of brain and ventral cord glia. (B) *vvl-41* drives reporter expression in a set of subesophageal ganglion (SOG) interneurons. (C-E) *vvl-43*, *-45* and *-46* activate expression in different subsets of ventral cord and/or brain neurons. (F) *vvl-48* drives expression in cells that line the tracheal tubes associated with the brain and ventral cord. (G) *vvl-49* activates reporter expression in CNS midline cells, presumably glia. (H and I) *vvl-50* and *-51* drive expression in subsets of brain and ventral cord neurons. (J) *vvl-53* activates reporter expression in brain and ventral cord NB lineages and in their neurons. (K) The *vvl-54* cluster drives reporter expression in subsets of brain and ventral cord neurons. (L) *vvl-55* activates reporter expression in a subset of both brain and ventral cord neurons. Based on the presence of membrane tagged GFP with in axons that exit the ventral cord, many of the neurons are most likely motor neurons.

**Figure 6:**
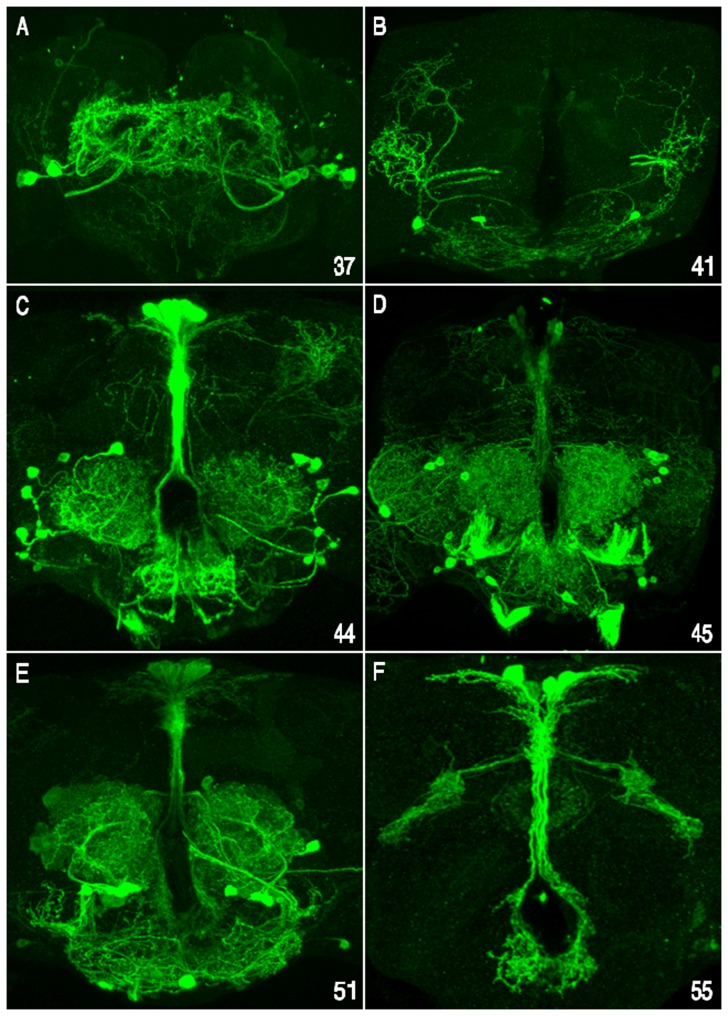
Expression of enhancer/reporters within the adult brain. Many of the tested conserved regions (six are illustrated) activate reporter expression in neurons or glia positioned within different sub-regions of the central brain. Shown are ventral (A) or anterior (B–F) views of adult brain. (A) *vvl-37* drives expression in several SOG neurons whose axons project across the midline. (B) *vvl-41* drives expression in several SOG neurons that project across the midline or dorsally. (C-F) *vvl-44*, *-45*, *-51* and *-55* all activate reporter expression in putative insulin-producing neurons (IPCs) [59] (C) *vvl-44* drives expression in IPCs and a set of lateral neurons whose dendrites fill the olfactory lobe. (D) *vvl-45* drives expression in a set of ventral brain neurons whose dendrites fill the olfactory lobe and the lateral brain. (E) *vvl-51* drives expression in IPCs and a set of ventral neurons whose axons and dendrites project into the olfactory lobe. (F) *vvl-55* drives expression in IPCs and in presumptive ellipsoid body neurons.

The dynamic expression of both of these genes may be regulated in part by multiple close-range enhancers that reside near their minimal promoters. Indeed, *vvl* has over 30 CSB clusters positioned within 25 kb of its transcribed DNA (Figure 1A; and data not shown). To assess the *cis*-regulatory nature of CSB clusters positioned beyond the local confines of these structural genes, we individually tested 19 consecutive conserved clusters within a 27 kb non-coding region (located 30 kb downstream of *vvl* and 57 kb from the 3′ end of *Prat2*) for their ability to regulate transgene reporter expression during embryonic development, in 3^rd^ instar larvae, and in the adult brain (Figures 4, 5, and 6). Each of the tested clusters (*vvl-37* to *vvl-55*, so named because of their proximity to *vvl*), coincides with phastcon peaks present in the UCSC genome browser conservation track (Figure 1B) [19]. Figure 2 shows a relaxed *EvoPrint* of five of the consecutive clusters (*vvl-37* to *vvl-41*) and highlights sequences that are conserved in all, or all but one, of the 12 species used in the analysis. A relaxed *EvoPrint* of the remaining clusters is shown in Figures S1, S2, S3, and S4. Super-blocks, multiple CSBs separated by conserved spacer lengths, are indicated by red-colored lowercase bases between CSBs (Figure 2). Also highlighted in the *EvoPrint* are prominent repeat sequences detected by *cis*-Decoder CSB cluster self-alignments (Figures 2 and S1, S2, S3, and S4).

We tested each of the CSB clusters independently using gypsy-insulated enhancer/reporter transgenes [17] [40]. To control for chromosomal integration-specific events that could influence reporter expression, we employed the *phiC31* mediated site-specific integration system to insure that all reporter transgenes were inserted into the same chromosomal environment [41] [42]. Remarkably, all 19 clusters functioned as spatial/temporal specific enhancers and 10 of these clusters generated distinct expression patterns in all three tested developmental phases (embryonic, larval and adult brain; summarized in Table 2 and Figures 4, 5, 6 and Text S1). In addition, five of the enhancers were active in just two of the developmental windows examined, while only four enhancers restricted their regulatory behavior to a single developmental phase.

**Table 2:** *cis*-Regulatory activity of consecutive *vvl* conserved sequence clusters.

Cluster	Embryo	Larva	Adult	Figure
*vvl-37*	Negative	Negative	Subset of brain neurons	6A
*vvl-38*	antenno-maxillary complex & anterior gut	Negative	negative	4A
*vvl-39*	PNS glia	Putative ventral cord glia	Putative glia	4B, 5A
*vvl-40*	Pair of cephalic lobe NBs	Negative	Putative glia	4C
*vvl-41*	CNS neuroblastlate lineage	Subset of brain neurons	Subset of brain neurons	4D, 5B, 6B
*vvl-42*	Posterior gut and ectoderm	Negative	Negative	4E
*vvl-43*	Late ectoderm	Ventral cord neurons	Subset of brain neurons	4F, 5C
*vvl-44*	Midline & PNS precursors	Negative	Central brain neurons and IPCs	4G, 6C
*vvl-45*	At stage 15, single neuron per cephalic lobe	Subset of brain and ventral cord neurons	Subset of central brain neurons and IPCs	4H, 5D, 6D
*vvl-46*	Gut	Ventral midline and brain neurons	Subset of brain neurons	4I, 5E
*vvl-47*	CNS and Neuroectoderm	A few cells in the SOG	Negative	4J
*vvl-48*	Trachea	Tracheal tubes associated with the brain & ventral cord	Optic lobe and central brain trachea	4K, 5F
*vvl-49*	Ventral cord midline glia	Ventral cord midline glia	Putative glia	4L, 5G,8
*vvl-50*	Negative	Subset of brain and ventral cord neurons	Brain and optic lobe neurons	5H
*vvl-51*	Ventral cord and PNS	Subset of brain and ventral cord neurons including motor neurons	Subset of Optic lobe, brain neurons and IPCs	4M, 5I, 6E
*vvl-52*	Anterior Tip	Negative	Negative	4N, 5J
*vvl-53*	Late temporal network NBs and neurons	Brain lineages including NBs and ventral cord neurons	Negative	4O
*vvl-54*	Gut ring	Subset of brain and ventral cord neurons	Subset of central Brain neurons	5K
*vvl-55*	placode cells or PNS neurons	motor neurons	Subset of central brain neurons and IPCs	4L, 6F

The majority of the reporter expressions observed were remarkably complex bilaterally symmetrical patterns that encompassed a diversity of cells types whose identities, in some cases, are unknown. Seventeen of the clusters activated unique pattern-specific expression during embryonic development in a wide diversity of tissues and cell types, including CNS neuroblasts (NBs), neurons and/or glia, PNS precursor cells, ectodermal cells and cells lining sub-regions of the gut and trachea (Figure 4; and Text S1). Fourteen of the clusters generated cell and/or region specific expression patterns within the 3^rd^ instar larva CNS (Figure 5; and data not shown), while thirteen drove reporter expression within overlapping sub-regions and/or cell types of the adult brain (Figure 6; and data not shown). The *cis*-regulation of adjacent enhancers exhibited unique non-overlapping dynamic expression patterns (Figure 4). In addition, *cis*-Decoder analysis revealed that each of the CSB clusters contained unique combinations of repeat and palindromic elements (Figures 2 and Figures S1, S2, S3, and S4). High-resolution views of each of the expression patterns illustrated in Figures 4, 5, and 6 are available at the *cis*Patterns website (http://cispatterns.ninds.nih.gov/). Although many of the enhancer expression patterns matched sub-patterns of *vvl* expression, we are unable to state with certainty that these enhancers regulate *vvl* expression.

### Species-specific variability in CSB spacing within enhancers

**Figure 7:**
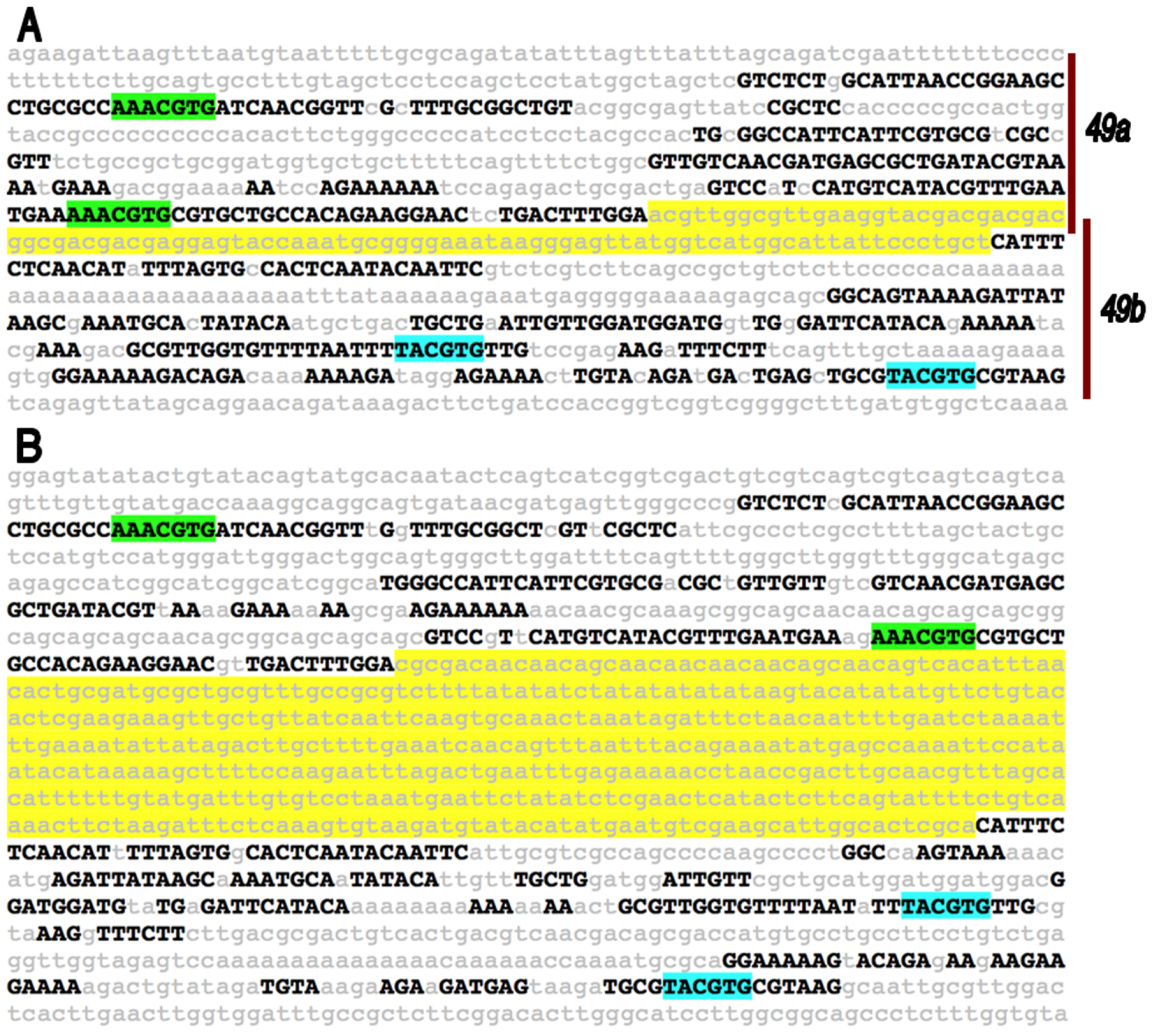
Species-specific flexibility within the *vvl-49* ventral cord midline enhancer. Twelve species *EvoPrint* analysis of the *vvl-49* CSB cluster reveals that its central non-conserved region has experienced a 466 bp insertion in *D. grimshawi* that is missing in the other drosophilids. (A) The *D. melanogaster* reference sequence *EvoPrint* of the *vvl-49* cluster. *cis*-Decoder analysis of *vvl-49* CSBs reveals four consensus Single-minded/Tango TF DNA-binding sites (ACGTG). Two different repeat elements were identified that contain different flanking repeat sequences (highlighted green and blue). The yellow highlighted 94 bp non-conserved region corresponds to the central *D. grimshawi* region shown in panel (B). Cluster sub-fragments (*49a* and *49b*) that were tested for enhancer activity are indicated by vertical bars on the left-margin. (B) An *EvoPrint* of the *vvl-49* CSB cluster using *D. grimshawi* as the reference sequence. The *EvoPrint* identified a 466 bp insertion (highlighted yellow) within the non-conserved central region (when compared to the *D. melanogaster EvoPrint*).

**Figure 8:**
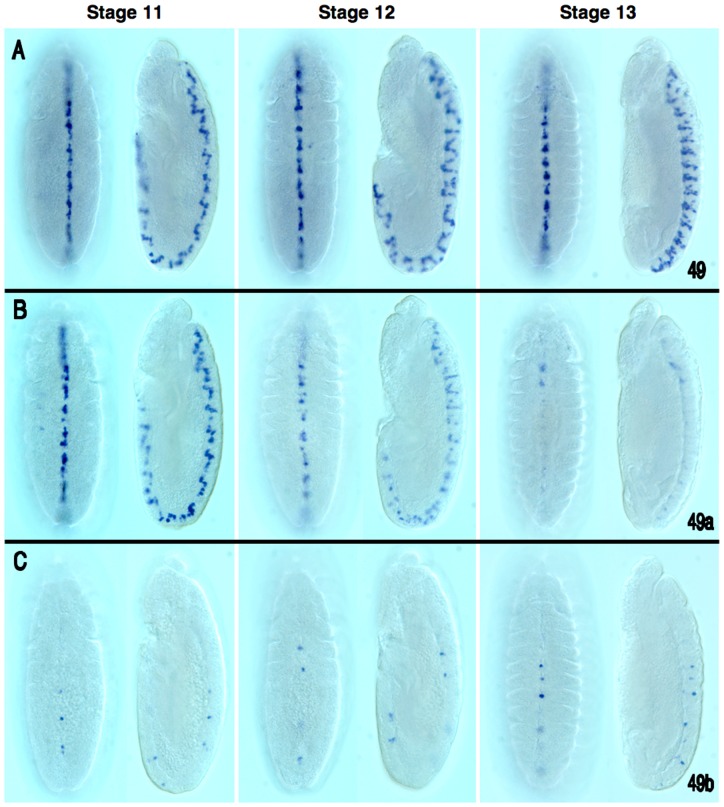
Expression analysis of *vvl-49* midline enhancer sub-domains. Temporal expression of *vvl-49* enhancer/reporter transgenes during embryonic development. (A) The entire *vvl-49* cluster drives reporter expression in a set of midline cells continuously from stages 11 through 13. (B) At stage 11, the *D. melanogaster vvl-49a* sub-fragment onsets expression in a subset of midline cells that is indistinguishable from that of the whole cluster, however, expression progressively declines in stage 12 and stage 13 embryos. (C) Expression of the *vvl-49b* sub-region activates expression in only a subset of the midline cells compared to the full *vvl-49* enhancer activity.

Our previous studies have shown that intervening non-conserved spacer regions between adjacent CSB clusters exhibit greater inter-species sequence length variability when compared to sequence length variability between CSBs within clusters [2]. Analysis of the spatial distribution of CSBs within the *vvl-49* cluster in different orthologous DNAs revealed that the *D. grimshawi* cluster has an additional 466 bp of non-conserved DNA within its central region that was not found in the other species (Figure 7). To determine if the *D. grimshawi* insertion indicated that the 1^st^ and 2^nd^ halves of the *vvl-49* cluster represent two closely spaced enhancers or semi-autonomous functional sub-domains of a single enhancer, we tested the corresponding *D. melanogaster* cluster halves for independent embryonic enhancer activity. The *vvl-49* enhancer activates transgene expression in a subset of ventral cord midline cells during embryonic stage 11 and expression persists throughout development (Figures 4, 6 and 7). Midline expression is most likely mediated via the midline cell-identity TFs Single-minded and Tango; their consensus DNA-binding site is present within four of the *vvl-49* CSBs with two binding sites residing in each cluster half (Figure 7). Figure 8 illustrates the temporal progression of the full *vvl-49* enhancer expression and the expression driven by the 1^st^ and 2^nd^ halves of the *D. melanogaster vvl-49* CSB cluster, *vvl-49a* and *vvl-49b* (see Figure 7A for CSB boundaries). Expression of the full enhancer was maintained from stage 11 through stage 15 (stages 11–13 illustrated in Fig. 8A). While the onset and timing of expression at stage 11 for the upper half of the cluster (*vvl-49a*) was essentially identical to the full cluster, subsequent reporter expression rapidly declined, so that by stage 13 there was only weak expression in midline cells (Figure 8B). In contrast, reporter expression driven by the lower half (*vvl-49b*) was detected in the ventral cord midline, but in considerably fewer cells than expression of the full cluster or its 1^st^ half (Figure 8C). Taken together, the different *cis*-regulatory behaviors of the upper and lower halves indicate that the upper half of the cluster may function to establish midline expression and the lower half to maintain full midline expression. Additional studies that address the function(s) of individual CSBs within *vvl-49* are required to understand how the two halves interact to maintain full enhancer activity.

## Discussion

The principal finding of this study is that many *Drosophila* intergenic regions and large introns are home to a remarkable diversity of *cis*-regulatory enhancers. Conservation tracks that span the *Drosophila* genome demonstrate that highly conserved clusters of CSBs are not confined to sequences within or adjacent to genes (Figure 1 and [17]). *EvoPrint* analysis of gene-distant or large intron genomic expanses reveal that most of the phastcon “peaks” [19] within these regions correspond to individual conserved clusters that are separated from each other by poorly conserved DNA (Figures 1B and 2). The evolutionarily constrained spacing of CSBs within these clusters and their high degree of repeat and palindromic element coverage (that frequently exceeds 60%) are both features of characterized enhancers and strongly suggests that many of these novel sequences also participate in transcriptional regulation. Based on these observations and our earlier work on identifying repeat and palindromic elements within non-coding *Drosophila* CSB clusters [17], we estimate that the *Drosophila* genome contains over 70,000 functionally distinct enhancers, and many of these are most likely multifunctional, capable of directing gene expression during different developmental phases and in different cell types.

The use of multiple enhancers to regulate different aspects of *Drosophila* gene expression is well documented for dynamically expressed developmental determinants such as segmentation and neural genes [2]–[4] [43] [44]. Our survey of genomic regions surrounding other well-characterized cell-identity determinants (Table 1) combined with the functional analysis of *vvl*-associated enhancers highlights a high level of *cis*-regulatory complexity acting on developmental determinants. These results suggest that the diversity of enhancers within the tested region is not an exception, in that large numbers of clustered CSBs are also found throughout the genome. Many genes contain large introns that harbor multiple CSB clusters, including genes constituting the Hox clusters (e.g., *Antp* and *Ubx*), the neural determinants *pdm-1* and *pdm-2*, the *gsb* genes, and other genes associated with enhancer fields such as *jing, engrailed*, *18 w*, and *ds* (unpublished observations and Table 1). It would appear that in many cases there is a requirement for a large number of multiple independent enhancers to regulate the different spatial/temporal expression dynamics of developmentally important genes. It has been proposed that remote enhancers interact with proximal promoter sequences by enhancer/promoter tethering (see for example [25] [45]; reviewed by [9]). Without further analysis, which we believe is beyond the scope of this study, it is uncertain whether the enhancers identified in this study regulate *vvl*, *Prat2*, or other more remote genes.

During the *cis*-Decoder analysis of the different *vvl*/*Prat2* associated enhancers, we noticed that groups of neighboring CSBs within clusters maintain a genus-invariant fixed length spacing of non-conserved DNA (Figure 2). Our analysis of other known enhancers indicates the evolutionarily constrained invariant spacing between subsets of enhancer CSBs (CSB super-blocks) is a common feature of the substructure of many enhancers. The fixed spacing can be explained in part by structural constraints between CSBs required for enhancer function (for example [46]). Linked associations of adjacent CSBs could also be due to fixed spatial requirements for interactions of different transcriptional regulators (see for example [47]), or for maintenance of structural integrity on a larger scale, for example in assembly of an enhanceosome (reviewed by [48]).

All of the CSB clusters analyzed in this study contain multiple distinct repeat sequence motifs and many of them contain characterized TF binding sites. Previous studies have highlighted the importance of multiplicity of TF binding sites for enhancer function (reviewed by [9]). For example, studies on Notch target enhancers have emphasized the importance of Su(H)/CSL dimerization in enhancer activation [49]. Similarly Hb target enhancers display consensus Hb sites in fixed configuration, suggesting that Hb likewise multimerizes on enhancers [47]. We have shown the importance of the presence of multiple bHLH DNA binding sites in the *nerfin-1* NB enhancer [40]. The need for multiple TF binding sites within enhancers is currently not understood. Their presence may augment enhancer regulatory strength, be required for redundant functions and/or enable multiple interactions with other TFs including non-DNA binding cofactors. Alternatively, these enhancers may contain multiple enhanceosomes each requiring the same TF to integrate the capacity for gene *cis*-regulation in cells undergoing different developmental programs.

Our earlier genome wide search for late temporal network NB enhancers highlighted the importance of shared and balanced sequence elements as signatures of functionally related enhancers [17]. This previous search for NB enhancers identified *vvl-41* as belonging to a family of enhancers based on the shared presence of POU homeodomain and bHLH TF binding sites, often in overlapping and adjacent juxtaposition within CSBs. This current study has identified the *vvl-53* NB enhancer as an additional member of this enhancer family as it also shares conserved repeat elements with the late temporal network NB enhancers (see Text S1).

Many of the enhancers described in this study are multifunctional, in that they drive expression in two or more temporal windows or in developmentally different cells. Previous studies have described multifunctional enhancers in *Drosophila* and have dealt with the question of how gene expression patterns evolve [50]. It was suggested that novel *cis*-regulatory functions evolve by employing the hidden activities of pre-existing regulatory sequences, indicating that only a few mutations are sufficient to modulate enhancer behavior. Evolutionary flexibility of enhancer sequence is also evidenced by the high divergence of DNA sequence and TF binding site position within the *sparkling* eye enhancer among the *Drosophila* species (reviewed by [51]). Our finding of a species-unique insertion in the *vvl-49* CSB cluster indicates some sequence flexibility between enhancer CSBs may not adversely affect their regulatory behavior; additional studies are required to understand the significance of this insertion. Some of the clusters analyzed in our study are missing one or more CSBs in the different species providing additional evidence of structural changes within these enhancers. For example, the 6th CSB (CCAAATACATAATTA) of the *vvl-43* enhancer is present in all *Drosophila* species examined in this study except for *D. willistoni* (Figure S1 and data not shown). However, the significance of the species-unique variations will only be understood by testing the effect of these changes on enhancer regulatory behavior.

The structural aspects of enhancers, including the presence multiple CSBs, their integration into super-blocks, and their content of repeat and palindromic elements, suggest that probing enhancer architecture is key to understanding the mechanism of their *cis*-regulatory behavior. Our findings are compatible with a flexible enhanceosome model where an enhancer's *cis*-regulatory activity is responsive to different combinations of TFs that are expressed in cells undergoing different developmental programs. These studies also suggest that sequence conservation within enhancers is the norm, and that this evolutionary phenotype can be used to delimit distinct enhancers and to identify important elements necessary for their function.

## Materials and Methods

### Comparative genomic analysis

Gene-poor regions within the *Drosophila* chromosomes were identified with the UCSC genome browser (http://genome.ucsc.edu) [19]. Using *Drosophila melanogaster* as the reference species, analysis was carried out as described previously, using a relaxed *EvoPrint* [52]. Integrity of CSB clustering defined in this manner was tested by EvoPrinting homologous regions of other species, particularly of *D. willistoni*, *D. virlis*, *D. mojavensis and D. grimshawi*.

To reveal adjacent CSBs separated by invariant spacing, *D. melanogaster* CSB clusters were submitted for *EvoPrint* analysis. Orthologous regions of *D. willistoni*, *D. virlis*, *D. mojavensis and D. grimshawi* genomic sequences were curated from the *EvoPrint* scorecard. Each species' genomic sequence was subject to *EvoPrint* analysis. Pair-wise alignments of the *D. melanogaster* EvoPrint against the other species' EvoPrints were performed using the Gene-wise DNA block aligner ([53]: http://www.ebi.ac.uk/Tools/Wise2/Dbaform.html), which was designed to reveal colinear-conserved blocks that are flanked by non-conserved sequences of varying lengths. Common blocks shared by all pair-wise alignments were termed super-blocks.

### Enhancer-reporter transgenes

Genomic CSB clusters, detected by *EvoPrint* analysis, were amplified according to procedures described previously [4]. Primer sequences for each genomic fragment are provided in Table S1. PCR-amplified genomic fragments were inserted into the Invitrogen pCRII-TOPO vector for sequence verification. To test their *cis*-regulatory activity, fragments were transferred into a modified pCa4B site-specific integration vector termed pBullfinch-Gal4 [4] [17]. All transgenes were integrated on the 3^rd^ chromosome at the attp2 integration site [42]. Details of the cloning steps and vector sequence are available upon request.

### Embryo *in situ* localization of mRNA

Embryo collection and fixation were performed according to the procedures described by [54]. For *in situ* hybridization detection of reporter expression, we used the Berkeley *Drosophila* Genome project embryo in situ hybridization protocol (http://www.fruitfly.org/about/methods/RNAinsitu.html) adapted for 1.6 ml Eppendorf tubes. Gal4 mRNA expression detected by a DIG probe, generated using a Roche protocol and reagents. Staining was visualized using anti-FITC Fab fragments coupled to alkaline phosphatase. After whole-mount *in situ* hybridization, embryos were viewed in 70% glycerol/30% phosphate-buffered saline (PBS), and photographed using a Nikon microscope equipped with Nomarski (DIC) optics. Embryo developmental stages were determined by morphological criteria [55]. All details are available upon request.

### Immunohistochemistry and confocal imaging of larval and adult brains

In order to visualize CSB cluster enhancer activity in the larval and adult CNS, our cluster/GAL4 enhancer reporter lines were crossed to the UAS-mCD8::GFP reporter line [56]. Larval CNS dissection was performed as described previously [56]; immunohistochemistry used a rabbit anti-GFP antiserum (1∶1,500, Invitrogen, San Diego, CA). Confocal imaging was performed using a Zeiss LSM710 and Plan-Apochromat objective 10× (numerical aperture = 0.45). Serial optical sections (1,024×1,024 pixel resolution) were taken at 1 µm intervals along the dorso-ventral axis. The confocal image stacks were analyzed using ImageJ software (NIH, Bethesda, MD). For analysis of adult brain, at least three flies of mixed genders were collected after hatching and used for the analysis. Brain dissection and immunohistochemistry were performed as described previously [57] using a rabbit anti-GFP (1∶300, Torrey Pines Biolabs, East Orange, NJ). Confocal imaging was performed using a Zeiss LSM510 META and plan Neofluar objective 40× (numerical aperture = 1.3). Serial optical sections (512×512 pixel resolution) were taken at 1 µm intervals along the rostro-caudal axis. The confocal image stacks were analyzed using Imaris (Bitplane, Zurich, Switzerland) software.

## Supporting Information

Figure S1 **Gene-distant conserved sequence clusters are made up of multiple conserved sequence blocks.** A *D. melanogaster* relaxed *EvoPrint* spanning 6.6 kb of the tested region that includes *vvl* clusters 42 through 46 (indicated by vertical bars in left margin). This genomic region is located between the *vvl* and *Prat2* genes. CSB clusters are resolved by their flanking less-conserved inter-cluster sequences. Capital letters represent bases in the *D. melanogaster* reference sequence that are conserved in all, or all but one, of the following orthologous regions within the *D. simulans*, *D. sechellia*, *D. erecta*, *D. yakuba*, *D. ananassae*, *D. pseudoobscura*, *D. persimilis*, *D. willistoni*, *D. virilis*, *D. mojavensis* and *D. grimshawi* genomes. Less or non-conserved DNA is shown as lower case gray letters. Colored highlighted sequences represent conserved repeat and/or palindromic elements discussed in the Text S1.
(TIF)

Figure S2 **Gene-distant conserved sequence clusters are made up of multiple conserved sequence blocks.** A *D. melanogaster* relaxed *EvoPrint* spanning 6.6 kb of the tested region that includes *vvl* clusters 46 through 50 (indicated by vertical bars in left margin). For additional information see legend for Figure S1.
(TIF)

Figure S3 **Gene-distant conserved sequence clusters are made up of multiple conserved sequence blocks.** A *D. melanogaster* relaxed *EvoPrint* spanning 6.6 kb of the tested region that includes *vvl* clusters 50 through 54 (indicated by vertical bars in left margin). For additional information see legend for Figure S1.
(TIF)

Figure S4 **Gene-distant conserved sequence clusters are made up of multiple conserved sequence blocks.** A *D. melanogaster* relaxed *EvoPrint* spanning 1 kb of the tested region that includes *vvl* clusters 54 and 55 (indicated by vertical bars in left margin). For additional information see legend for Figure S1.
(TIF)

Table S1 **PCR primers used to clone** ***vvl*** **-37 through** ***vvl*** **-55 enhancers.**
(DOC)

Text S1 ***cis*** **-Regulatory behavior and structural analysis of 19 consecutive gene-distant CSB clusters.**
(DOC)
