# Supplementary figures and images for: cis-Regulatory Complexity within a Large Non-Coding Region in the Drosophila Genome
Source: PLoS One. 2013 Apr 22;8(4):e60137. doi: 10.1371/journal.pone.0060137 (PMC3632565; doi:10.1371/journal.pone.0060137)

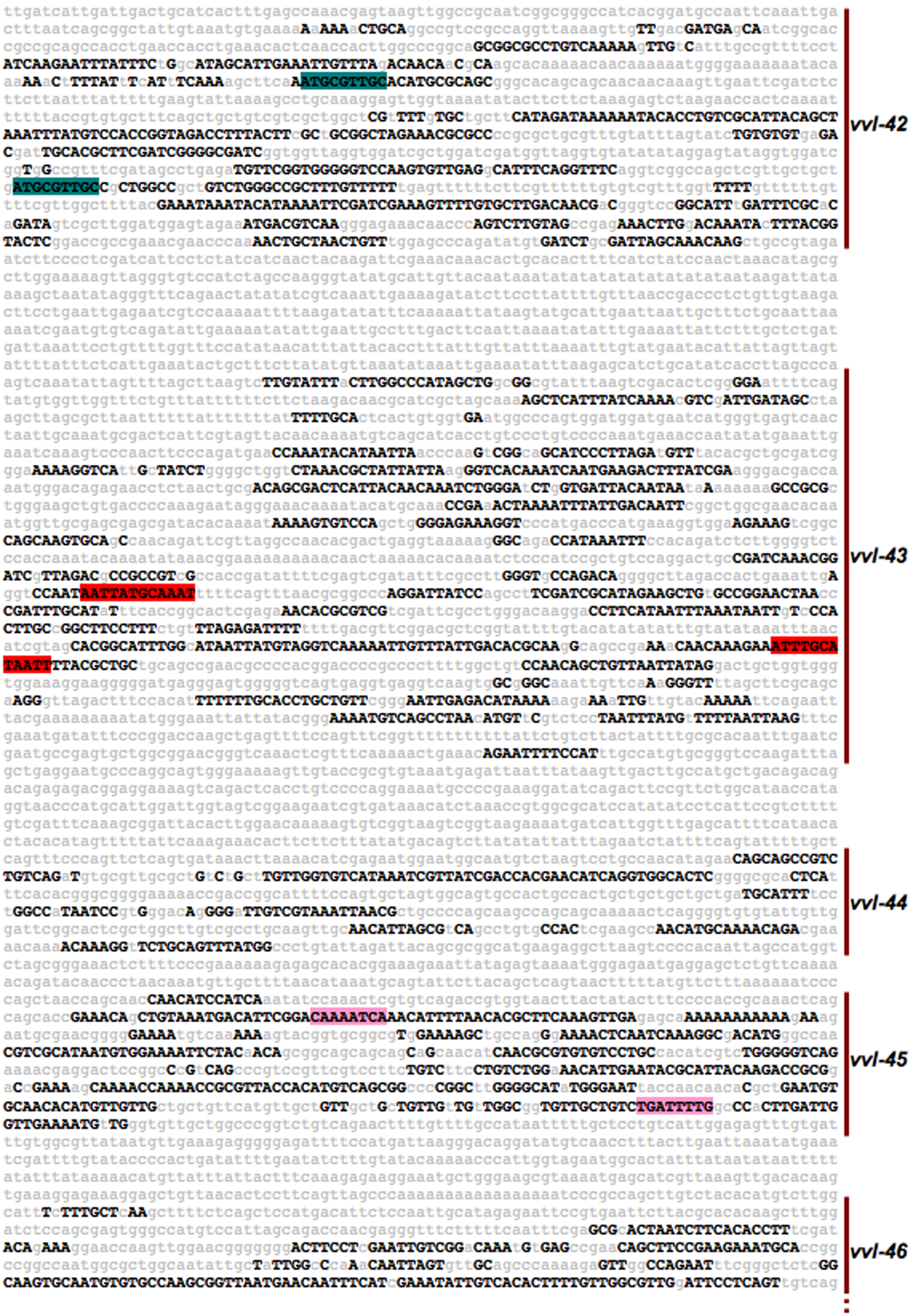

Supplement: Figure S1 — Gene-distant conserved sequence clusters are made up of multiple conserved sequence blocks. A D. melanogaster relaxed EvoPrint spanning 6.6 kb of the tested region that includes vvl clusters 42 through 46 (indicated by vertical bars in left margin). This genomic region is located between the vvl and Prat2 genes. CSB clusters are resolved by their flanking less-conserved inter-cluster sequences. Capital letters represent bases in the D. melanogaster reference sequence that are conserved in all, or all but one, of the following orthologous regions within the D. simulans, D. sechellia, D. erecta, D. yakuba, D. ananassae, D. pseudoobscura, D. persimilis, D. willistoni, D. virilis, D. mojavensis and D. grimshawi genomes. Less or non-conserved DNA is shown as lower case gray letters. Colored highlighted sequences represent conserved repeat and/or palindromic elements discussed in the Text S1. (TIF) [file pone.0060137.s001.tif]

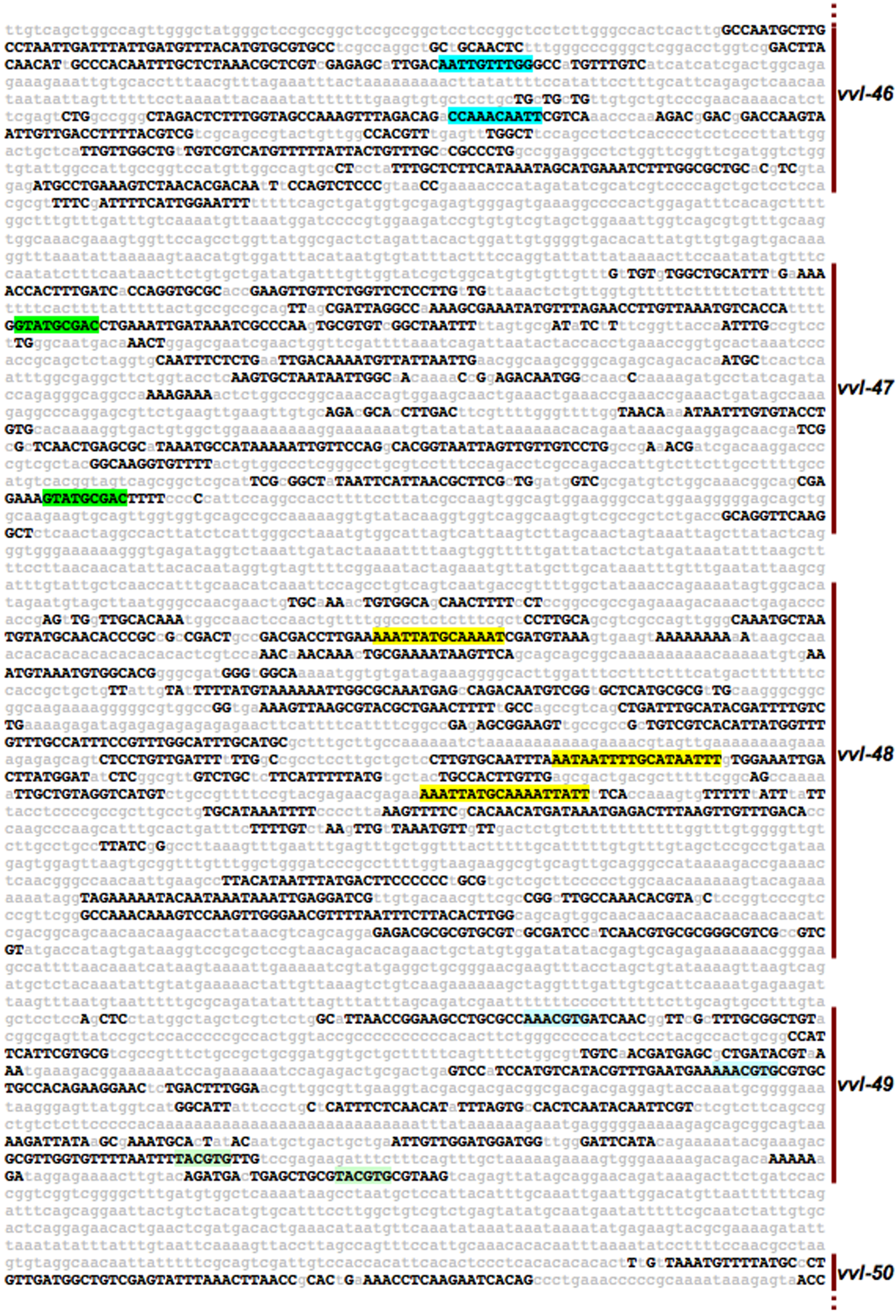

Supplement: Figure S2 — Gene-distant conserved sequence clusters are made up of multiple conserved sequence blocks. A D. melanogaster relaxed EvoPrint spanning 6.6 kb of the tested region that includes vvl clusters 46 through 50 (indicated by vertical bars in left margin). For additional information see legend for Figure S1. (TIF) [file pone.0060137.s002.tif]

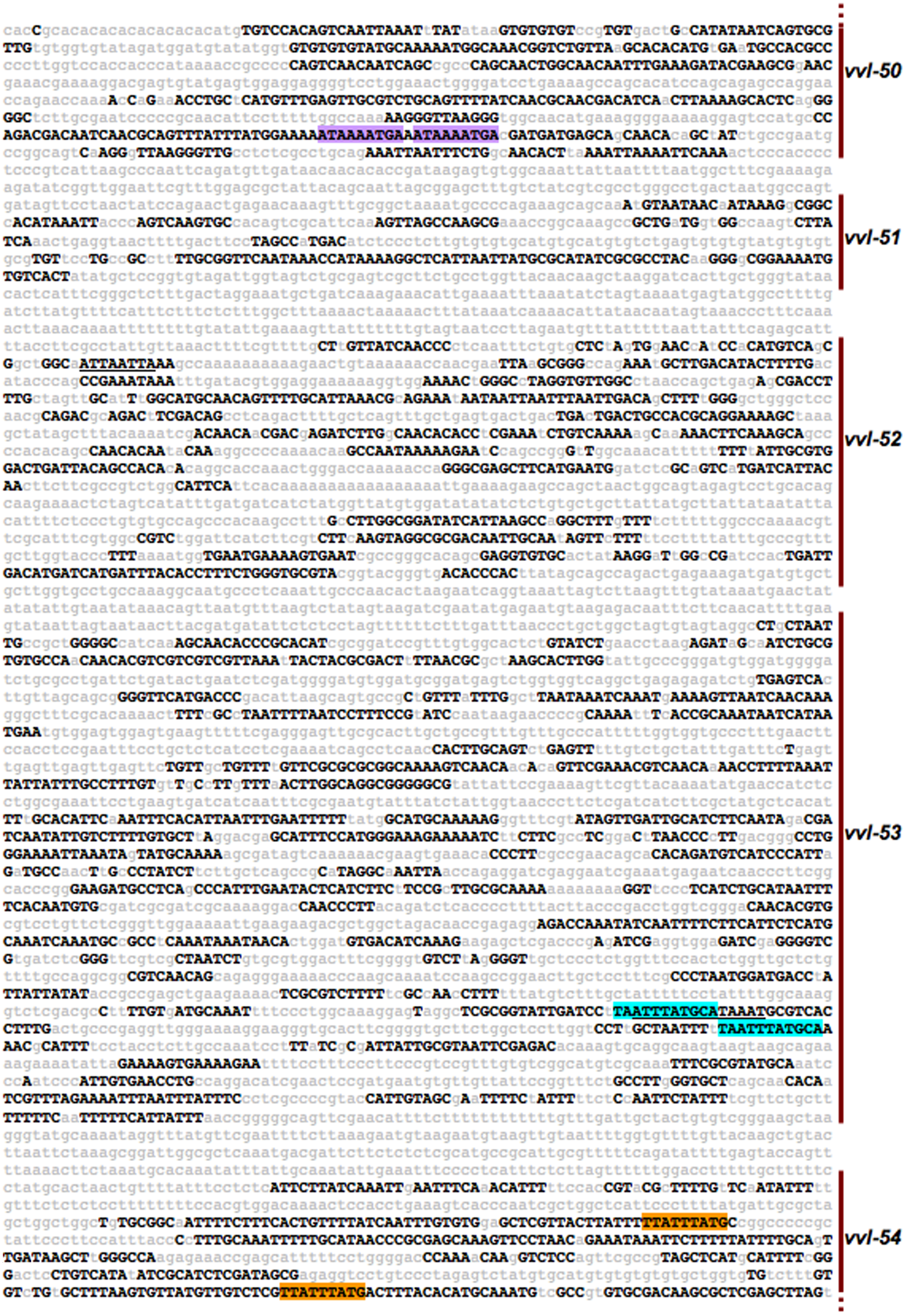

Supplement: Figure S3 — Gene-distant conserved sequence clusters are made up of multiple conserved sequence blocks. A D. melanogaster relaxed EvoPrint spanning 6.6 kb of the tested region that includes vvl clusters 50 through 54 (indicated by vertical bars in left margin). For additional information see legend for Figure S1. (TIF) [file pone.0060137.s003.tif]

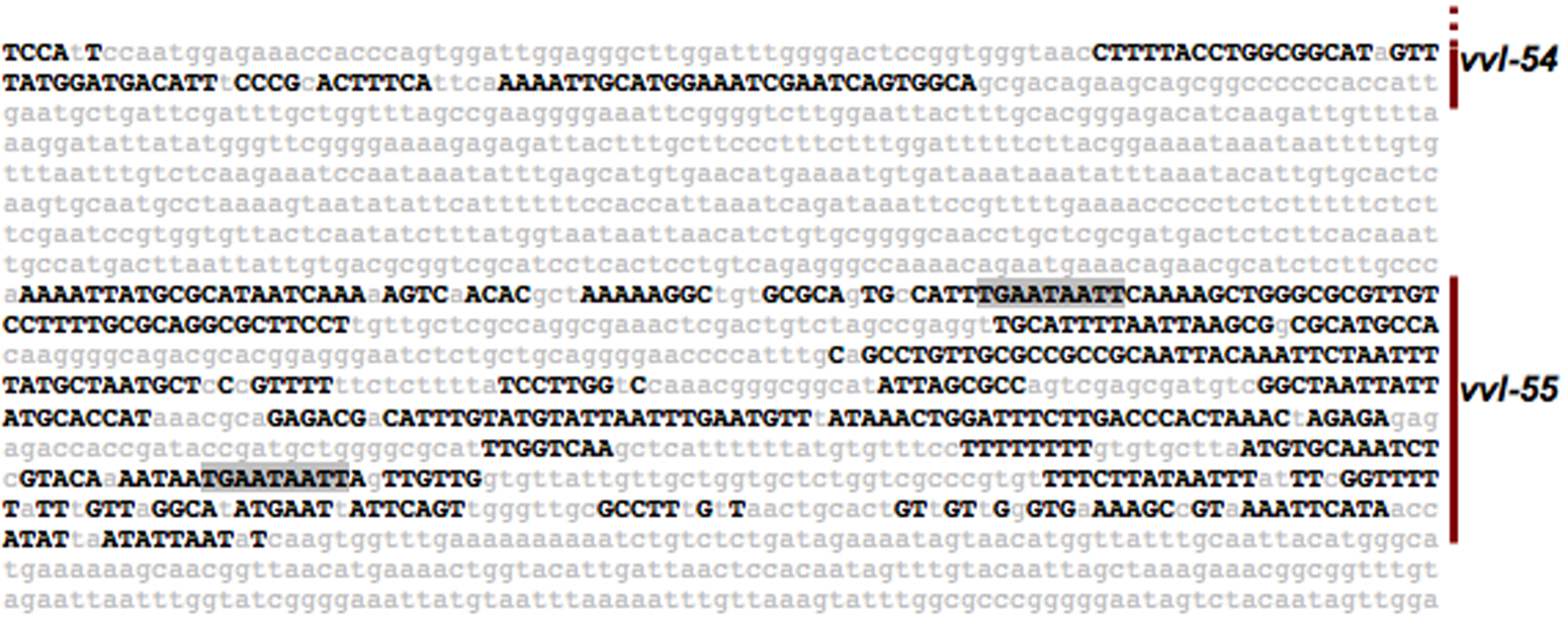

Supplement: Figure S4 — Gene-distant conserved sequence clusters are made up of multiple conserved sequence blocks. A D. melanogaster relaxed EvoPrint spanning 1 kb of the tested region that includes vvl clusters 54 and 55 (indicated by vertical bars in left margin). For additional information see legend for Figure S1. (TIF) [file pone.0060137.s004.tif]
