# Supplementary material for: cis-Regulatory Complexity within a Large Non-Coding Region in the Drosophila Genome
Source: PLoS One. 2013 Apr 22;8(4):e60137. doi: 10.1371/journal.pone.0060137 (PMC3632565; doi:10.1371/journal.pone.0060137)
Supplement: Table S1 — PCR primers used to clone vvl -37 through vvl -55 enhancers. (DOC) [file pone.0060137.s005.doc]

**Table S1. PCR primers used to clone *vvl*-37 through *vvl*-55 enhancers**

| **Cluster** | **5’ Primer sequence*** | **3’ primer (complementary sequence)**** |
| --- | --- | --- |
| *vvl*-37 | ctgcgtgaaatcttctgctc | cgcttaactgccttaagaagttt |
| *vvl*-38 | cctgcaatttggttctctcctgc | gggaacgagcaggatagtgagg |
| *vvl*-39 | ccctgtgcattgccaaggtc | gcttgatatgggctacacagg |
| *vvl*-40 | ccgttgccgtaacatttCCC | gggtcttgagagggttaagag |
| *vvl*-41 | gctatcttaaattcttgcca | ggatggaatgggtggtggaag |
| *vvl*-42 | ctgcatcactttgagccaaacg | ccctcgatcattcctctatc |
| *vvl*-43 | gcatatcaccttagcccaag | gcgggtccaagatttagctg |
| *vvl*-44 | cctgccaacatagaaCAGC | ccctgtattagattacagcg |
| *vvl*-45 | ctaaccagcaaccAACATCC | gctcctgtcattggagagtttg |
| *vvl*-46 | cttgtctacacatgtcttggc | cccactggagatttcacagcttttg |
| *vvl*-47 | gatttgttggtatcgctggc | gccaccttttccttatcgccaag |
| *vvl*-48 | agcctgtcagtcaatgaccg | ggtccgcgctccgtaacaga |
| *vvl*-49 | Cttgcagtgcctttgtagctcc | GCGTAAGtcagagttatagcagg |
| *vvl*-50 | ccaccacattcacactccc | ccgtcattaagcccaattcag |
| *vvl*-51 | cagaactgagaacaaagtttgcg | cggtgtagattggtagtctg |
| *vvl*-52 | cagagcattttaccttcgcc | gccagactgagaaagatgatg |
| *vvl*-53 | cctgctggctagtgtagtaggc | ggattggcgctcaaatgacg |
| *vvl*-54 | cctatgcactaactgttttatttcc | cccaccattgaatgctgattcg |
| *vvl*-55 | aacagaacgcatctcttgcc | caacggttaacatgaaaactgg |

* For cloning, an AscI restriction site was added the 5’-end.

** For cloning, a single restriction site (NheI, BglII or NheI) was added the 5’-end.
