# Supplementary material for: cis-Regulatory Complexity within a Large Non-Coding Region in the Drosophila Genome
Source: PLoS One. 2013 Apr 22;8(4):e60137. doi: 10.1371/journal.pone.0060137 (PMC3632565; doi:10.1371/journal.pone.0060137)
Supplement: Text S1 — cis -Regulatory behavior and structural analysis of 19 consecutive gene-distant CSB clusters. (DOC) [file pone.0060137.s006.doc]

**Supplemental Information**

**Text S1: *cis*-Regulatory behavior and structural analysis of 19 consecutive gene distant CSB clusters**

The following is a description of the structure and enhancer-reporter expression profiles of 19 CSB clusters within a 27 kb non-coding region (located 30 kb downstream of *vvl* and 57 kb from the 3’ end of *Prat2*). Although many of the enhancer expression patterns match sub-patterns of *vvl* expression, we are unable to state with certainty that these enhancers regulate *vvl* expression, as Prat2 is also expressed in embryos and larvae [1].

***vvl-37*:** In the adult brain, *vvl-37* activates reporter expression in a subset of ventrally positioned neurons that appear to be part of antennal mechanosensory and motor centers [2]. The membrane tagged CD8-GFP reporter expression reveals projection of neurites contralaterally across the midline of the subesophageal ganglion (SOG) (Figure 6A). *vvl-37* reporter expression was not detected during embryonic or larval development. *EvoPrint* analysis revealed that the *vvl-37* cluster contained a total of 288 conserved bases arrayed in 18 CSBs (Figure 2). DNA block alignments revealed six super-blocks that included 13 CSBs. Self-alignment of the CSBs using the *cis*-Decoder CSB aligner [3] revealed the presence of 14 distinct repeat sequence elements covering 47% of the CSBs (data not shown).

***vvl-38*:** During embryonic stage 13, *vvl-38* activates expression in a small set of antennomaxillary complex cells and within a restricted region of the anterior gut (Figure 4A). No expression in the larval or in the adult brain was detected. In *D. melanogaster* *vvl-38* spans 838 bp, and consists of 12 CSBs containing 398 conserved base pairs (Figure 2). *cis*-Decoder identified 41 repeat elements that covered 75% of the conserved sequence. The longest conserved repeat (AAAAATTATT) is present twice on a single CSB (Figure 2, highlighted blue). The most frequent repeat is a hexamer (ATTGTT) appearing five times. When a flanking C was included in the analysis, a heptamer (CATTGTT) occurred three times (data not shown).

***vvl-39*:** Embryonic expression is restricted to a subset of segmentally arrayed small cells in the ectoderm of stage 13 embryos (Figure 4B). Due to the lack of a fixed segmental pattern observed among multiple embryos of the same developmental stage, their position within the ectoderm and the later expression of this enhancer (see below), we speculate that the cells are most likely migrating glial or phagocytic cells (Figure 4B and data not shown). Larval expression was detected in glial cells that line the ventral nerve cord, and in the adult in many putative central brain glia (Figure 5a and data not shown). The enhancer cluster spans 1.1 kb in *D. melanogaster* of which 490 bp are conserved (Figure 2). *cis*-Decoder self-alignment of its 31 CSBs identified 28 distinct repeat elements that covered 66% of the conserved sequence (data not shown). The longest conserved repeat, appearing in a single super-block, is a nonamer (CAAGTGGCA; yellow highlight in Figure 2).

***vvl-40*:** During embryonic development, *vvl-40* reporter expression is restricted to two adjacent NBs within each cephalic lobe, with expression persisting through embryonic stage 11 (Figure 4C). The *vvl-40* cluster represents the smallest cluster in this genomic region (Figure 2). It spans 561 bases, and consists of 8 CSBs containing a total of 139 conserved bases. *cis*-Decoder identified 3 repeat elements that cover 25% of the conserved sequence. One of the repeat sequences consists of a hexamer (TAATTG). ***vvl-41*:** In the developing embryonic CNS *vvl-41* drives reporter expression in NBs during late lineage development in the brain and ventral cord and transiently in ventral cord midline cells (Figure 4D and [3]). In the third instar larval CNS, *vvl-41* activates expression in a subset of SOG interneurons that project their neurites dorsally into the brain and into anterior-posterior longitudinal axon fascicles of the ventral cord (Figure 5B). In addition, a bilaterally symmetrical pair of thoracic ventral cord neurons expresses the reporter. In the adult, reporter expression is expressed in a small number of brain neurons that project across the SOG and into the ventrolateral protocerebrum (Figure 6B). The *vvl-41* CSB cluster spans 1,495 bp in *D. melanogaster*, and contains of 38 CSBs representing 675 conserved bases (Figure 2). *vvl-41* contains 10 super-blocks incorporating 19 of its CSBs. Self-alignment of CSBs identifies 61 distinct repeat sequence elements that cover 63% of the conserved sequence (data not shown). A previous study identified *vvl-41* as a late temporal network NB enhancer based on its sharing of sequence elements with the late temporal network *cas-6* NB enhancer [3]*.*  *cis*-Decoder analysis reveals that *vvl-41* shares two POU-domain TF octamer binding sites (ATGCAAATG; highlighted purple in Figure 2) with the *cas-6* enhancer (data not shown).

***vvl-42*:** Enhancer/reporter activity is restricted to embryonic development. Transgene expression was detected in epithelial cells that line the anal pad and isolated ectodermal cells of unknown identity (Figure 4E). No larval or adult expression was discernable. The *vvl-42* cluster spans 1,242 bp in *D. melanogaster* and consists of 23 CSBs with a total of 450 bp (Figure S1). *cis*-Decoder analysis identified 26 repeat elements that represent 50% of its conserved sequence (data not shown). A prominent repeat present in this cluster is ATGCGTTGC, highlighted teal blue in Figure S1. Of note, a composite *e*-BLAT [4] between *D. melanogaster* and the *D. willistoni* orthologous region revealed the 5’ -> 3’ orientation of the *vvl-42* and *vvl-43* clusters are inverted in *D. willistoni* compared with the other 11 *Drosophila* species (data not shown).

***vvl-43*:** This cluster activates reporter expression within ectodermal cells of the thoracic and abdominal segments during stage 13 (Figure 4F and [3]). In the larval CNS, bilateral expression was detected in a subset of thoracic and abdominal neurons (Figure 5C). Based on their axonal track projections that exit the CNS, most appear to be motor neurons. Adult expression was detected in a set of ventral neurons that project dorsally and then across the midline (data not shown). The *vvl-43* cluster spans 1,763 bp in *D. melanogaster* and consists of 41 CSBs with a total of 663 conserved bases (Figure S1). *cis*-Decoder analysis of its CSBs identified 42 distinct repeat elements that covered 61% of the conserved sequence (data not shown). Among the repeats identified by *cis*-Decoder is a 12mer (AATTATGCAAAT, highlighted red in Figure S1). The first six bases of this 12mer (AATTATG) are repeated six times in the cluster.

***vvl-44*:** During embryonic development, reporter expression is detected in a single ventral cord midline cell per segment and in putative PNS precursor cells (Figure 4G and data not shown). No expression was detected in larvae. In the adult, the *vvl-44* activates expression in insulin-producing cells (IPCs) (Figure 6C). Dendritic arbors were apparent in the antennal lobe and within the SOG. Prominent expression was also detected in large dorsal medial putative neurosecretory neurons. The *vvl-44* cluster spans 387 bases and consists of 154 conserved bases constituting 11 CSBs (Figure S1).

***vvl-45*:** Expression of the enhancer reporter transgene in embryos was detected in a single bilateral pair of cells in the brain at stages 14-15 (Figure 4H). In the third instar larval CNS, a complex pattern of expression was observed in presumptive neurons (judging from their large cell bodies) in the brain, SOG and ventral cord, including motor neurons (Figure 5D). Similarly in the adult CNS, expression was found in a complex pattern of neurons, notably in IPCs, and in the olfactory lobe and SOG (Figure 6D). The membrane-tagged CD8-GFP was also detected a large central brain dendritic arbor. Expression in the optic lobe was observed in tangential neurons of the medulla and lobula (data not shown). The *vvl-45* cluster spans 611 bases in *D. melanogaster* and consists of 15 CSBs with a total of 371 conserved bases (Figure S1). *cis-*Decoder self-alignment program revealed the presence of 20 repeat and palindromic sequence elements covering 66% of the conserved bases (data not shown). Notable is the repeat sequence CAAAATCA, highlighted pink in Figure S1. *EvoPrint* alignments revealed that both *vvl-45* and *vvl-46* are inverted in *D. willistoni*.

***vvl-46*:** Enhancer/transgene reporter expression was detected in the posterior midgut at stage 10 and in both the anterior and posterior midgut segments at stage 14 (Figure 4I and data not shown). No expression was detected in central portion of the midgut. Larval CNS expression was detected in a population of bilaterally symmetrical putative interneurons in both the brain and central cord (Figure 5E). In the adult central brain, expression was detected in ventral positioned neurons that project anteriorly (data not shown). *vvl-46* spans 1.2 kb in *D. melanogaster* and consists of 25 CSBs with a total of 501 conserved bases (Figures S1-2). *cis*-Decoder analysis identified 39 RPS elements that covered 60% of the conserved sequence. Prominent among the repeat sequences is the presence of two decameric elements (CCAAACAATT: Figure S2; blue highlight).

***vvl-47*:** During embryonic development, *vvl-47* activates reporter expression in a small cluster of cells per hemisegment within the lateral ectoderm and ventral cord during stage 11 (Figure 4J). In the larva it is expressed in a bilaterally symmetrical cluster of cells in the ventral brain, and in a small group of cells in the anterior part of the adult brain (data not shown). *EvoPrint* analysis revealed that *vvl-47* is inverted in *D. virilis, D. mojavensis*, and *D. grimshawi* relative to the other species. *vvl-47* cluster spans 1.2 kb in *D. melanogaster* and consists of 20 CSBs with a total of 345 conserved bases (Figure S2). *cis*-Decoder alignments revealed 18 RPS elements covering 48% of the conserved clusters (data not shown). Prominent among the repeat sequences in *vvl-47* is a nonamer (GTATGCGAC) present in two CSBs (green highlight in Figure S2).

***vvl-48*:** The onset of embryonic expression was first detected in segmentally repeated clusters of cells at stage 11 (Figure 4K) that appear to be associated with the tracheal placodes (reviewed by [5]). Ectodermal expression is maintained through embryonic stage 14. From stage 12 onward reporter expression initiates and persists in the dorsal tracheal trunk. Larval and adult expression was also detected in cells that make up the trachea (Figure 5F and data not shown). *vvl-48* extends across 1.7 kb and consists of 41 CSBs and 850 conserved bases (Figure S2). *cis*-Decoder alignment revealed the presence of 65 repeat elements covering 80% of the CSBs (data not shown). The cluster contains two 18mers (AATAATTTTGCATAATTT, highlighted yellow). An additional sequence (AAATTATGCAAAAT, also highlighted in yellow) matches 14 of the 18 bp in this repeat.

***vvl-49*:** The *vvl-49* reporter is expressed in a subset of ventral cord midline cells starting at embryonic late stage 11 and continues through stage 15 (Figure 4L and 8A; and data not shown). Based on the position of cells along axon fascicle commissure tracks and on cell morphology of the reporter-positive cells at stage 15, these cells are most likely glia (data not shown). *vvl-49* expression was detected in ventral cord midline cells and putative brain glia of larvae (Figure 4G), and in a few large neurons of the anterior ventral region of the adult brain that project posteriorly and form a dendritic arbor in the SOG (data not shown). The cluster spans 797 bp in *D. melanogaster* and contains 16 CSBs with a total of 311 conserved bases (Figure 7a and Figure S2). *cis*-Decoder alignment revealed 17 repeat elements covering 41% of the conserved bases. There are four consensus-binding sites for the midline TFs Single-minded/Tango heterodimer (ACGTG) [6], two of which have flanking As (highlighted light blue) and two of which have a flanking T (highlighted light green). We report on the further functional analysis of this enhancer in the main part of the paper.

***vvl-50*:** Reporter expression was not detected in the embryo, however, prominent expression was seen in larval brain and ventral cord motor neurons (Figure 5H). Expression was also detected in a set of neurons in the adult brain (data not shown). *vvl-50* cluster extends over 774 bp, and consists of 21 CSBs made up of 431 conserved bases (Figures S2-3). Self-alignment revealed 40 repeat elements that covered 68% of the CSB sequences (data not shown). Prominent among the repeat sequences is a nonamer (ATAAAATGA, highlighted purple) appearing on the same CSB.

***vvl-51*:** Embryonic expression of the *vvl-51* enhancer/reporter was evident in segmentally repeated putative PNS neurons during embryonic stage 13 (Figure 4M). Larval expression of *vvl-51* was detected in neurons of the brain and ventral cord, including motor neurons projecting axons from thoracic segments (Figure 5I). Other prominent features of the larval pattern included reporter expression in a SOG dendritic arbor and ventral cord neurons that project contralaterally, crossing the ventral midline. Expression was detected in the adult brain in a set of posterior neurons and another set of large anterior neurons that are most likely IPCs (Figure 6E). Expression was also detected in neurons that project to the optic lobe medulla (data not shown). *vvl-51* extends over 276 bp and includes 8 CSBs with a total of 149 conserved bases (Figure S3).

***vvl-52*:** Starting at embryonic stage 14, the enhancer/reporter transgene was expressed in a bilaterally symmetrical pair of cells within the antennomaxillary complex (Figure 4N). No expression was detected in the larval or adult CNS. The *vvl-52* cluster extends over 1,239 bp and has 27 CSBs consisting of 395 conserved bases (Figure S3). Self-alignment showed that *vvl-52* contains 32 distinct repeat elements that covered 58.5 % of the conserved sequences (data not shown).

***vvl-53*:** During embryonic development, the *vvl-53* reporter is expressed in a subset of CNS NBs during late lineage development and in their ganglion mother cells, both in the brain and ventral cord (Figure 4O). In 3^rd^ instar larvae, reporter expression was detected in cephalic lobe neural precursor cells, their neurons, and within ventral cord precursor cells that generate interneurons (Figure 5J). Examination of earlier larval stages reveals that each lineage contains a large diameter cell surrounded by smaller cells, indicating that the enhancer drives expression in NBs during lineage development, with continued expression in neurons (Figure 5J and data not shown). Expression in the adult CNS was detected in the mushroom body (data not shown). This large cluster spans 2,442 bp and consists of 65 CSBs that constitute 908 conserved bases (Figure S3). *cis*-Decoder self-alignment revealed 120 distinct repeats, which cover 78% of the conserved bases. Prominent among the repeats is a 14 bp palindrome (ATTTATGCATAAAT; underlined). There are two copies of the undecamer (TAATTTATGCA, highlighted blue). Our earlier study identified NB enhancers that drove transgene expression during late phases of NB lineage development [3]. *cis*-Decoder comparative analysis revealed that these enhancers also share multiple repeat and palindromic elements with *vvl-53*. For example, both *cas-6* and *vvl-53* enhancers contain overlapping homeodomain and POU-domain TF DNA-binding sites in identical configurations (TAATTTATGCAAA).

***vvl-54*:** Embryonic expression of the *vvl-54* reporter transgene is found within in a ring of cells that make up a sub-region of the anterior gut at stage 14 (data not shown). Expression in the larva CNS was detected in a set of thoracic neurons; expression in the adult brain was found in subsets of bilaterally symmetrical neurons (Figure 5K; and data not shown). The cluster spans 725 bp and consists of 21 CSBs (Figures S3-4). *cis-*Decoder alignment shows the presence of 34 distinct repeat elements covering 58% of the conserved sequences (data not shown). The cluster contains a nonameric repeat element (TTATTTATG; Figure S3, highlighted orange).

***vvl-55*:** During embryonic development, reporter expression was detected in segmentally repeated cells that line the lateral trunks of the developing trachea (Figure 4P). *vvl-55* activates expression in larvae in a subset of ventral cord motor neurons and is expressed in IPCs and putative ellipsoid body neurons (Figure 5L). Expression in the adult is in putative anterior neurosecretory cells whose neuritis project ventrally towards the SOG and laterally. The *vvl-55* cluster extends over 692 bp of genomic sequence and contains 20 CSBs with a total number of 400 conserved bases (Figure S4). Self-alignment revealed the presence of 38 repeats covering 66% of the conserved sequence (data not shown). *vvl-55* contains two copies of the nonamer TGAATAATT (gray highlighted sequence).

**Text S1 References**

1. Tomancak P, Beaton A, Weiszmann R, Kwan E, Shu S, et al. (2002) Systematic determination of patterns of gene expression during *Drosophila* embryogenesis. Genome Biol 3: RESEARCH0088.

2. Homberg U, Christensen TA, Hildebrand JG (1989) Structure and function of the deutocerebrum in insects. Annu Rev Entomol 34: 477-501.

3. Brody T, Yavatkar AS, Kuzin A, Kundu M, Tyson LJ, et al. (2012) Use of a *Drosophila* genome-wide conserved sequence database to identify functionally related cis-regulatory enhancers. Dev Dyn 241: 169-189.

4. Yavatkar AS, Lin Y, Ross J, Fann Y, Brody T, et al. (2008) Rapid detection and curation of conserved DNA via *enhanced*-BLAT and *EvoPrinterHD* analysis. BMC Genomics 9: 106.

5. Zelzer E, Shilo BZ (2000) Cell fate choices in *Drosophila* tracheal morphogenesis. Bioessays 22: 219-226.

6. Wharton KA, Jr., Franks RG, Kasai Y, Crews ST (1994) Control of CNS midline transcription by asymmetric E-box-like elements: similarity to xenobiotic responsive regulation. Development 120: 3563-3569.
